# MicroRNAs as potential targets for progressive pulmonary fibrosis

**DOI:** 10.3389/fphar.2015.00254

**Published:** 2015-11-05

**Authors:** Subbiah Rajasekaran, P. Rajaguru, P. S. Sudhakar Gandhi

**Affiliations:** Department of Biotechnology, Bharathidasan Institute of Technology Campus, Anna UniversityTiruchirappalli, India

**Keywords:** idiopathic pulmonary fibrosis, miRNAs, inflammation, TGF-β1, epithelial cells, fibroblasts, α-SMA, type-1-collagen

## Abstract

Idiopathic pulmonary fibrosis (IPF) is a chronic, progressive and devastating disorder. It is characterized by alveolar epithelial cell injury and activation, infiltration of inflammatory cells, initiation of epithelial mesenchymal transition (EMT), aberrant proliferation and activation of fibroblasts, exaggerated deposition of extracellular matrix (ECM) proteins, and finally leading to the destruction of lung parenchyma. MicroRNAs (miRNAs) are endogenous small non-coding RNA molecules that post-transcriptionally regulate gene expression in diverse biological and pathological processes, including cell proliferation, differentiation, apoptosis and metastasis. As a result, miRNAs have emerged as a major area of biomedical research with relevance to pulmonary fibrosis. In this context, the present review discusses specific patterns of dysregulated miRNAs in patients with IPF. Further, we discuss the current understanding of miRNAs involvement in regulating lung inflammation, TGF-β1-mediated EMT and fibroblast differentiation processes, ECM genes expression, and in the progression of lung fibrosis. The possible future directions that might lead to novel therapeutic strategies for the treatment of pulmonary fibrosis are also reviewed.

## Introduction

Idiopathic pulmonary fibrosis (IPF) is a chronic and progressive fibrosing lung disorder (Gross and Hunninghake, 2001; Wynn, 2007), which is the first or second most commonly encountered lung disease (17–86%) among various interstitial lung diseases (ILD) in clinical settings (Coultas and Hughes, 1996; Karakatsani et al., 2009; Ley and Collard, 2013; Musellim et al., 2014). However, its overall incidence and prevalence is unclear due to geographic or demographic differences in the risk of IPF (Ley and Collard, 2013). The median survival period of IPF patients is 2–3 years from the time of diagnosis (Raghu et al., 2011). Despite the fact that the etiology of IPF is largely unknown, in some patients, abnormalities in genes such as surfactant protein A2 (SFTPA2), surfactant protein C (SFTPC), ELMO/CED-12 domain containing 2 (ELMOD2), mucin 5b (MUC5B), and two telomerase genes (hTERT and hTR) are considered as risk factors for pulmonary fibrosis (Khalil et al., 2001; Hodgson et al., 2006; Seibold et al., 2011; Armanios, 2012). Similarly, some environmental factors, namely cigarette smoking, viral infections and exposure to metal and wood dust are also considered as risk factors (Hubbard et al., 1996; Baumgartner et al., 1997; Kelly et al., 2002). Several studies have reported that IPF likely results from alveolar epithelial injury and subsequent dysregulated repair process (Horowitz and Thannickal, 2006; Wilson and Wynn, 2009). The pathological hallmarks of IPF include recruitment of inflammatory cells and excessive secretion of pro-fibrotic mediators, such as transforming growth factor-β1 (TGF-β1), and platelet-derived growth factor (PDGF), aberrant activation of epithelial mesenchymal transition (EMT), fibroblasts activation and proliferation, and persistence of apoptotic resistant myofibroblasts in the lesions (Todd et al., 2012; Samarakoon et al., 2013).

During the active period of fibrosis in humans and animal models, the presence of myofibroblasts in fibrotic lesions is amply documented (Adler et al., 1989; Mitchell et al., 1989; Kuhn and McDonald, 1991; Pache et al., 1998). Studies show that myofibroblasts express α-smooth muscle actin (α-SMA), a stress fiber and are considered to be the cells responsible for the deposition of the extracellular matrix (ECM) that constitutes the scar (Kuhn and McDonald, 1991). Myofibroblasts have the potential of intensifying or prolonging the inflammation associated with fibrosis (Flavell et al., 2008) and also have contractile property that is thought to be important in wound contraction (Adler et al., 1989). Although fibroblasts are well-documented progenitor cells for myofibroblasts, recent studies suggested that fibrocytes (Quan et al., 2004), pericytes (Hung et al., 2013), epithelial (Liu, 2004) and endothelial (Piera-Velazquez et al., 2011) cells are also the candidate precursors of myofibroblasts. The successful healing process is associated with the gradual disappearance of myofibroblasts, however, their continued presence results in the fibrogenic cytokine expression, exaggerated production and deposition of ECM in the lung parenchyma, and subsequent impaired tissue regeneration or pathologic fibrosis (Kuhn and McDonald, 1991). In recognition of the potential importance of myofibroblasts in fibrosis, studies are focused on the nature and precise roles of this cell type in the context of pulmonary fibrosis.

Further, sustained dysregulation in ECM homeostasis alone can result in life-threatening pathological conditions as the increased ECM synthesis, accumulation and subsequent cross-linking may lead to altered biochemical and biomechanical matrix properties (Lu et al., 2011). Although constitutive activation of collagen-secreting myofibroblasts is reported to be responsible for increasing collagen secretion and accumulation, an imbalance of matrix mettalloproteinases (MMPs) and their inhibitors, the tissue inhibitors of metalloproteinases (TIMPs) has been shown to contribute in the incomplete matrix remodeling and irreversible fibrosis (Lu et al., 2011). MMPs are zinc-dependent, secreted or cell surface based endopeptidases, and centrally involved in the turnover of ECM components such as collagens and proteoglycans. MMPs activity is tightly regulated at several levels, including transcription and translation, compartmentalization, and inhibition by their endogenous inhibitors, the TIMPs (Lemaitre and D’Armiento, 2006). In addition, other matrix metalloproteinases, a disintegrin motifs (ADAMTS) families, and serine proteinases, which include plasmin and cathepsin G are specialized in degrading the ECM. Other proteinases like cysteine proteinase, aspartate proteinase, and threonine proteinase are predominantly active at acidic pH and mainly digest intracellular proteins (Cawston and Young, 2010). In contrast, the cysteine proteases namely, cathepsins B and L can be secreted outside the cell and digest ECM as well (Green and Lund, 2005). Hence, understanding of ECM components and factors involved in ECM remodeling in pulmonary fibrosis is also crucial for uncovering novel therapeutic targets and treatment strategies. In spite of some progress made to understand the development of severe pulmonary fibrosis, current therapeutic options such as corticosteroids alone or in combination with immunosuppressive drugs such as cyclophosphamide, azathioprine, and colchicine are available with limited success. Therefore, it is important to have a recent update on understanding of cellular and regulatory factors for presenting novel therapeutic strategies against lung fibrosis.

In this context, recently, MicroRNAs (miRNAs), a growing family of small non-coding RNAs, have gained significant attention for their work as post-transcriptional regulators of gene expression and control various cellular processes such as differentiation, proliferation, and cell-cell interaction (Foshay and Gallicano, 2007; Bueno et al., 2008; Inui et al., 2010). In addition, miRNAs dysregulations are linked to a wide spectrum of diseases, including proliferative vascular disease, cardiac disorders, kidney diseases, diabetes mellitus, fibrosis and cancer (Thum et al., 2008; Kato et al., 2009; Lee and Dutta, 2009; Kumar et al., 2012; Noetel et al., 2012; Zampetaki and Mayr, 2012). Thus, this review emphasizes recent knowledge on specific miRNAs that are differentially expressed in human IPF lungs and also describes pro-fibrotic or anti-fibrotic role of specific miRNA in animal models of lung fibrosis. Further, those miRNAs that directly regulate pro-inflammatory mediators, EMT, fibroblast proliferation and differentiation, TGF-β signaling, and ECM gene expression (Type-1-collagen) (COL1) are also taken into consideration. Finally, the review concludes with suggestions on strategies for testing miRNAs that could lead to the development of new clinical tools in IPF therapy.

## miRNAs: Biogenesis and Biological Functions

MicroRNAs are encoded in the genomes of plants and animals either by intergenic or intragenic locations. miRNA biogenesis begins in the nucleus, where they are initially transcribed by RNA polymerase II or, in a few cases by RNA polymerase III as a capped and polyadenylated transcript, known as primary miRNA (pri-miRNA) (Du and Zamore, 2007). Pri-miRNAs are processed by the RNase III endonuclease, Drosha, and its cofactor, DiGeorge syndrome critical region 8 (DGCR8) into 60–100 nt stem-looped structures known as precursor miRNAs (pre-miRNAs) (Denli et al., 2004; Han et al., 2004). Pre-miRNAs are then transported to the cytoplasm by Exportin-5, where they are further processed by a second RNase III, Dicer, producing 25 nt mature miRNA duplexes (miRNA–miRNA^+^) (Murchison and Hannon, 2004; Chendrimada et al., 2005). The miRNA^+^ strand is degraded, whereas the miRNA-strand is preferentially retained and loaded into the RNA-induced silencing complex (RISC) containing enzymes of the Argonaute (AGO-2) family. The enzyme complex binds to the 3′-untranslated region (UTR) of target mRNA, depending on the degree of complementarity, gene silencing occurs through either inhibition of translation or degradation of its target mRNAs (Bartel, 2009). While miRNAs are generally considered as repressors of gene expression, they have exceptionally been reported to stimulate mRNA translation. For instance, Vasudevan et al. (2007) showed that human miR-369-3 directs an association of Argonaute (AGO) and fragile X mental retardation-related protein 1 (FXR1) with AU-rich elements (AREs) of tumor necrosis factor alpha (TNF-α) to initiate translation upon cell cycle arrest. Further, two miRNAs, namely let-7 and the synthetic microRNA miRcxcr4 likewise induce translation upregulation of target mRNAs upon cell cycle arrest. Similarly, Henke et al. (2008) reported that miR-122 induced translation of hepatitis C viral RNA, suggesting the requirement of more research to understand the miRNA-mediated regulation of translation.

## miRNAs in Idiopathic Pulmonary Fibrosis: Humans and Animal Models

This section deals with the expression of dysregulated miRNAs in patients with IPF (see summary in **Table 1**) as well as the role of specific miRNA in the pathogenesis of pulmonary fibrosis in animal models (see summary in **Table 2**). In animal models, although several agents such as bleomycin, silica, fluorescein isothiocynate, radiation and viral vectors (Degryse and Lawson, 2011) are used to recapitulate human IPF, each has its own merits and demerits (Degryse and Lawson, 2011). Bleomycin-induced pulmonary fibrosis has been widely used and is a well characterized model in rodents to understand the molecular mechanisms involved in fibrogenesis and for the evaluation of potential therapies (Moore and Hogaboam, 2008). Moreover, some of the histological features of human IPF, including collagen deposition and myofibroblast differentiation are comparable to bleomycin-induced pulmonary fibrosis in mice. Hence, most of the published literature described herein used bleomycin-induced lung injury in a mouse model for studying the role of miRNAs in experimental settings.

**Table 1:** Dysregulated microRNAs (miRNAs) in lungs of patients with idiopathic pulmonary fibrosis (IPF).

S. NO	Upregulated miRNA	Downregulated miRNA	Validation method	Tissue/cell type	Reference
1	miR-409-3p, miR-92b, miR-376a, miR-205, miR-31, miR-765, miR-199b, miR-198, miR-622, miR-330, miR-379, miR-659, miR-182, miR-487b, miR-299-5p, miR-127, miR-296, miR-509, miR-557, miR-134, miR-491, miR-132, miR-155, miR-99a, miR-324-3p, miR-214, miR-199a, miR-320	let-7d, miR-125a, miR-126, miR-138, miR-17-3p, miR-184, miR-197, miR-203, miR-224, miR-26a, miR-30a-3p, miR-30a-5p, miR-30b, miR-30c, miR-30d, miR-338, miR-362 and miR-92	microRNA micro arrays	Lungs	Pandit et al., 2010

2	miR-21		*In situ* hybridization	Lungs	Liu et al., 2010b

3	miR-21	miR-200c	*In situ* hybridization and qRT-PCR	Alveolar type II cells of lungs	Yamada et al., 2013

4		miR-200a and c	qRT-PCR	Lungs	Yang et al., 2012

5	miR-31, miR-31^∗^, miR-493^∗^, miR-76a, miR-382, miR-127-3p, miR-410, miR-376c, miR-432, miR-377, miR-654-3p, miR-409-3p, miR-381, miR-299-5p, miR-1, miR-487b, miR-133b, miR-370, miR-513c, miR-299-3p, miR-543, miR-369-5p, miR-154, miR-1225-5p, miR-409-5p, miR-379, miR-650, miR-143^∗^, miR-495, miR-513a-5p, miR-143, miR-214^∗^, miR-411, miR-199b-5p, miR-199a-5p, miR-199b-3p, miR-376a^∗^, miR-27b, miR-539, miR-585, miR-509-5p, miR-10a^∗^ and miR-509-3-5p	miR-33a^∗^, miR-151-3p, miR-361-3p, miR-181a, miR-374b, miR-425, miR-222, miR-532-3p, miR-17, let-7d, miR-668, miR-30c-1^∗^, miR-548c-3p, miR-532-5p, miR-362-5p, miR-342-5p, miR-181b, miR-885-5p, miR-181a^∗^, miR-517b, miR-520g, miR-628-3p, miR-340^∗^, miR-744^∗^, miR-652, miR-502-3p, miR-29b-1^∗^, miR-30a^∗^, miR-30a, miR-30d, miR-7-1^∗^, miR-500^∗^, miR-181d, miR-210, miR-30c-2^∗^, miR-224, miR-30b^∗^, miR-223, miR-221^∗^, miR-126, miR-502-5p, miR-375, miR-522, miR-598, miR-326, miR-489, miR-223^∗^, miR-30b, miR-203, miR-338-3p and miR-184	microRNA micro arrays	Lungs	Milosevic et al., 2012

6		miR-29	qRT-PCR	Lungs	Montgomery et al., 2014

7		miR-375	qRT-PCR	Lungs	Wang et al., 2013

8	miR-199a-5p		qRT-PCR and *In situ* hybridization	Selectively increased in fibrotic foci of lungs	Lino Cardenas et al., 2013

9		miR-17 ∼ 92 cluster	qRT-PCR and *In situ* hybridization	Lungs and fibroblasts	Dakhlallah et al., 2013

10		miR-326	qRT-PCR	Lungs	Das et al., 2014

11	miR-96		qRT-PCR and *In situ* hybridization	Lungs and fibroblasts	Nho et al., 2014

12		miR-338-3p, miR-126-3p, miR-30d-5p, miR-203a, miR-30b-5p, miR-30a-5p, miR-101-3p, miR-126-5p, miR-375, miR-218-5p, miR-92a-3p, miR-222-3p, miR-26b-5p, miR-125a-5p, miR-138-5p, miR-184, miR-26a-5p, miR-326, miR-211-5p, miR-598-3p, miR-452-3p, miR-643, miR-621, miR-512-5p, miR-569, miR-33a-3p, miR-581, miR-517b-3p, let-7d-5p and let-7g-5p	MicroRNA micro arrays	Lungs	Berschneider et al., 2014

13		miR-26a	qRT-PCR	Lungs	Liang et al., 2014b

14	miR-382, miR-449a, miR-642, miR-205, miR-31, miR-34b, miR-376b and miR-376c	miR-378, miR-422a, miR-425, miR-497, miR-500, miR-660, miR-663, miR-744, miR-92a, miR-93, miR-101, miR-103, miR-106a, miR-106b, miR-1271, miR-130a, miR-130b, miR-138, miR-141, miR-150, miR-15a, miR-15b, miR-17, miR-181b, miR-181c, miR-181d, miR-184, miR-185, miR-18a, miR-191, miR-193b, miR-194, miR-197, miR-19a, miR-19b, miR-203, miR-20a, miR-20b, miR-22, miR-221, miR-222, miR-223, miR-23a, miR-24, miR-25, miR-26a, miR-27a, miR-29a, miR-29b, miR-29c, miR-30a, miR-30b, miR-30c, miR-30d, miR-30e, miR-320a, miR-320b, miR-320c, miR-320d, miR-335, miR-345 and miR-375	microRNA micro arrays	Lungs	Liang et al., 2014a

15	miR-210		*In situ* hybridization	Lungs	Bodempudi et al., 2014

*^∗^indicates passenger strand.*

**Table 2:** miRNAs linked to pulmonary fibrosis in animal models.

S. NO	miRNA	Model	Species	Putative role	Reference
1	Let-7d	Inhibition	Mouse	Anti-fibrotic	Pandit et al., 2010
2	miR-21	Bleomycin	Mouse	Pro-fibrotic	Liu et al., 2010b
3	miR-200c	Bleomycin	Mouse	Anti-fibrotic	Yang et al., 2012
4	miR-29	Bleomycin	Mouse	Anti-fibrotic	Xiao et al., 2012; Montgomery et al., 2014
5	miR-17 ∼ 19 cluster	Bleomycin	Mouse	Anti-fibrotic	Dakhlallah et al., 2013
6	miR-145	Bleomycin	Mouse	Pro-fibrotic	Yang et al., 2013a
7	miR-326	Bleomycin	Mouse	Anti-fibrotic	Das et al., 2014
8	miR-26a	Bleomycin	Mouse	Anti-fibrotic	Liang et al., 2014a,b
9	miR-98	Bleomycin	Rat	Anti-fibrotic	Gao et al., 2014

The let-7 family of miRNAs was one of the first discovered (Reinhart et al., 2000) and extensively studied in metastasis. Pandit et al. (2010, 2011) for the first time reported the implication of a member of the let-7 family in a non-tumor disease. They found that let-7d expression was mainly localized to the alveolar epithelium in normal lungs, but was significantly decreased in IPF lungs. They also established that let-7d inhibition caused a significant downregulation of epithelial markers such as *E*-cadherin (E-CAD), and tight junction protein-1 (TJP-1) and a significant upregulation in the expression of mesenchymal markers such as COL1, non-histone chromosomal high-mobility group 2 (HMGA2), and α-SMA in bleomycin-induced lung injury mice model. Similarly, miR-21 was first identified as an oncogenic miRNA in targeting many tumor suppressor genes, including phosphatase and tensin homolog (PTEN) (Pan et al., 2010). However, its expression is found to be highly upregulated in myofibroblasts of IPF lungs (Liu et al., 2010b). Whereas, Yamada et al. (2013) reported the upregulated expression of miR-21 occurred in the lung epithelial cells as well as in the cells surrounding fibrotic foci of human IPF lungs. It was also observed that miR-21 antisense probes attenuated collagen deposition as well as fibronectin (FN), α-SMA and COL1 expression in mice with bleomycin-induced pulmonary fibrosis (Liu et al., 2010b).

Several studies showed that miR-200 promotes EMT in cancerous cells (Gregory et al., 2008; Park et al., 2008; Yang et al., 2011). Recent investigation on the role of miR-200 family members in pulmonary fibrosis provided evidence that miR-200a and miR-200c were significantly downregulated in the lungs of mice with experimental pulmonary fibrosis and in the lungs of patients with IPF (Yang et al., 2012). In support of the above findings, Yamada et al. (2013) illustrated decreased expression of miR-200c in the lungs of IPF patients. Introduction of miR-200c into the mice lungs diminished experimentally induced pulmonary fibrosis as elicited by lowered lung collagen content and α-SMA expression, suggesting its anti-fibrotic role (Yang et al., 2012). Further, a recent study using miRNA microarray expression revealed that several miRNAs were differentially expressed in the lungs of IPF patients when compared to that of the control lungs (Milosevic et al., 2012). Among 43 over expressed miRNAs, 24 were localized to the microRNA cluster on chromosome 14q32, and 13 of them were members of the miR-154 family (Milosevic et al., 2012). Although the role of miR-154 in the animal models of pulmonary fibrosis is unknown, the pro-fibrotic role of miR-154 family was confirmed *in vitro* by analyzing the proliferation and differentiation of lung fibroblasts (Milosevic et al., 2012).

Downregulation of miR-29 family members correlated with many types of cancer and fibrosis (Calin et al., 2005; Cushing et al., 2011; Roderburg et al., 2011). However, Xiao et al. (2012) established the therapeutic potential of miR-29 for pulmonary fibrosis. The Sleeping Beauty (SB) transposon-mediated gene transfer of miR-29b prevented bleomycin-induced pulmonary fibrosis in mice as demonstrated by reduced Masson’s trichrome staining, hydroxyproline content, COL1, type-3-collagen (COL3) and FN expression. Further, miR-29b was able to suppress major fibrotic factors such as TGF-β1 and connective tissue growth factor (CTGF) as well as phosphorylation of SMAD3. In support of this finding, Montgomery et al. (2014) observed a comparable decline in the levels of miR-29 family members (miR-29a, miR-29b, and miR-29c) in the lung biopsies of patients with IPF and also a significant decrease in hydroxyproline content and reduced trichrome staining in miR-29b mimic-treated mice when compared to bleomycin-instilled mice. These data indicated that miR-29 mimic could be a potent therapeutic miRNA for treating pulmonary fibrosis.

miR-375 is a pancreatic islet-specific miRNA as it regulates insulin secretion and pancreatic islet development (Kloosterman et al., 2007; Liu et al., 2010a). Recently, lowered expression of miR-375 was observed in severe human IPF lungs when compared with less severe IPF and control samples (Wang et al., 2013). However, the role of miR-375 in animal models of pulmonary fibrosis is unknown. Previously, miR-199-5p has been linked to the inner ear hair cell development and chondrogenesis and also in tumor progression (Friedman et al., 2009; Lin et al., 2009). In the context of lung fibrosis, high expression of miR-199-5p was selectively observed in fibrotic foci of human IPF lungs (Lino Cardenas et al., 2013). This finding was further confirmed by performing miRNA microarray assay in bleomycin-treated mice lungs. In addition, *in situ* hybridization assay performed in bleomycin-injured lungs revealed, selective expression of miR-199-5p in myofibroblasts. Ectopic expression of miR-199-5p promoted the pathogenic activation of pulmonary fibroblast including proliferation, invasion, migration and differentiation. Thus, due to its selective overexpression in fibrotic foci, miR-199-5p could be considered as a potential target in the development of novel therapies to treat pathological lung fibrosis.

The miR-17∼92 cluster is critical for lung epithelial cell homeostasis and development. Mice lacking this cluster have only few epithelial cells and die from asphyxia at birth (Ventura et al., 2008). miR-17∼92 expression was lower in the lung biopsies from patients with IPF when compared to the control patients (Dakhlallah et al., 2013). Its expression was also lower in the lungs of bleomycin treated mice. However, treatment with 5′-aza-2′-deoxycytidine, a demethylating agent significantly enhanced the expression of miR-17∼92 cluster leading to significantly reduced Masson’s trichrome staining for collagen and expression of fibrotic genes such as COL1a1, collagen-13a1 (COL13a1), vascular endothelial growth factor (VEGF), CTGF, and DNA methyltransferases -1 (DNMT-1). This study also suggested the existence of a novel epigenetic feedback loop between miR-17∼92 and DNMT-1 in lung fibrosis.

Yang et al. (2013a) compared the development of bleomycin-induced lung fibrosis in wild-type and *miR-145^-/-^* mice and found that *miR-145^-/-^* mice lungs had diminished collagen deposition, reduced expression of α-SMA and increased expression of kruppel-like factor 4 (KLF4), a negative regulator of α-SMA, suggesting pro-fibrotic role of miR-145. miR-326 was the first identified miRNA with high expression in neurons with anti-neuronal effects (Nye et al., 1994). In the context of IPF, its expression was found to be downregulated in IPF patients when compared to control samples (Das et al., 2014). Further, miR-326 administration to mice with bleomycin-induced pulmonary fibrosis caused a significant downregulation of TGF-β1, matrix metalloproteinase-9 (MMP-9), ETS-1 (v-ets avian erythroblastosis virus E26 oncogene homolog 1), and SMAD3 phosphorylation and a significant increase in SMAD7 expression (Das et al., 2014). These results suggested that miR-326 plays an anti-fibrotic role by regulating TGF-β1 and other pro-fibrotic gene expression.

Nho et al. (2014) demonstrated the occurrence of miR-96 positive cells in the fibroblastic foci in IPF patients. miR-96 is reported to directly bind to 3′-UTR of FOXO3a (Forkhead box O3) mRNA and subsequently inhibits its translation. However, the role of miR-96 on experimentally induced pulmonary fibrosis are yet to be explored. Similarly, Berschneider et al. (2014) reported that 30 miRNAs were significantly downregulated in IPF tissue specimens. Previous report suggested that WINT1-inducible signaling pathway protein 1 (WISP1) is a highly expressed pro-fibrotic mediator in IPF (Konigshoff et al., 2008). Overexpression of miR-30a/d and miR-92 downregulate TGF-β1-induced WISP1 expression in human lung fibroblasts without any effect on the expression of fibrotic genes such as COL1 and FN. However, *in vivo* role of miR-92 in animal models of pulmonary fibrosis remains unknown.

miR-26 is reported to play a significant role in growth, development, cell differentiation, tumor and non-tumor diseases (Gao and Liu, 2011). However, the expression of miR-26a was found to be downregulated in the lungs of mice with experimental pulmonary fibrosis and in IPF patients (Liang et al., 2014b). In mice, intratracheal instillation of miR-26a prior to bleomycin administration, significantly alleviated the exaggerated deposition of collagen, hydroxyproline content and expression of genes such as COL1, COL3, metalloproteinase-2 (MMP-2), MMP-9, CTGF and SMAD4. Following the above findings, the same research group showed that the inhibition of miR-26a induced mesenchymal markers expression, including vimentin (VIM) and α-SMA following bleomycin instillation (Liang et al., 2014a), confirming that the loss of function of miR-26a could facilitate lung epithelial cells to transform into myofibroblasts and induce pulmonary fibrosis in mice. miR-210 is a unique hypoxamir and regulates many vital functions including cell proliferation in response to hypoxia (Fasanaro et al., 2008). Increased miR-210 expression was observed in patients with rapidly progressive IPF (Bodempudi et al., 2014). The direct role of miR-210 on experimentally induced pulmonary fibrosis *in vivo* need to be investigated. Finally, the role of miR-98, another member of let-7 family was established in bleomycin-induced pulmonary fibrosis in rats (Gao et al., 2014). The study revealed that miR-98, can regulate STAT3-related signals and expression of genes such as α-SMA, COL1 and apoptotic factors (BAX/BCL2) thereby preventing pulmonary fibrosis. Thus, the above studies are indicative of the profound role of miRNAs in the onset and progression of pulmonary fibrosis.

## Roles of miRNAs on Pro-Inflammatory Mediators in Experimental Pulmonary Fibrosis

In the development of IPF, innate and adaptive immune systems appear to play vital roles (Luzina et al., 2008; Lafyatis and Farina, 2012). Cells of the innate or non-specific immune system (e.g., macrophages, neutrophils) are predominant but T-cells (adaptive, or specific immune system) are also major constituent in most IPF patients (Harrison et al., 1991; Wynn, 2008). Macrophages are activated by Th1 cytokine interferon-γ (INF-γ) and also by Th2 cytokines interleukin-4 (IL-4) and interleukin-13 (IL-13), acquiring pro-fibrotic phenotype (Capelli et al., 2005). When pulmonary inflammation and fibrosis occur, excessive accumulation of T-lymphocytes are diffusely present throughout the lung (Chizzolini, 2008). There is evidence that molecules derived from pathogenic organisms, paracrine signals derived from activated lymphocytes as well as autocrine factors produced by fibroblasts can cooperate to initiate and maintain myofibroblasts activation (Wynn, 2008). In animal models of fibrosis also proved that T-cells that lack CD28, a central constimulatory cell surface molecule that is necessary for full T-cell activation, revealed significantly diminished pulmonary fibrosis (Okazaki et al., 2001). Similarly, B-cells have also been implicated in the pathogenesis of pulmonary fibrosis, either by secreting interleukin-6 (IL-6) or by producing autoantibodies (Hasegawa et al., 2005).

Further, analysis of serum samples and lung biopsies from IPF patients contained more inflammatory cytokines such as TNF-α and in addition, mice that overexpress this cytokine in the lungs developed pulmonary fibrosis (Miyazaki et al., 1995). According to the current literature, a total of 9 miRNAs has been related to animal pulmonary fibrosis (see summary in **Table 2**) out of which only miR-29b has been shown to regulate the innate immune response *in vivo* (Montgomery et al., 2014). Significantly lower concentrations of interleukin-12 (IL-12), interleukin-14 (IL-14), and granulocyte colony-stimulating factor (G-CSF) were found in bronchoalveolar lavage (BAL) fluids from the lungs of mice treated with a combination of bleomycin and miR-29b mimic when compared to mice treated just with bleomycin. Additionally, bleomycin-induced elevation of BAL inflammatory cells was also brought down by miR-29b mimic treatment, indicating the inhibitory effect of miR-29b on the immune response.

## Roles Of miRNAs On Tgf-β1-Mediated Fibrogenic Signaling In Lung Epithelial Cells And Fibroblasts

**FIGURE 1:**
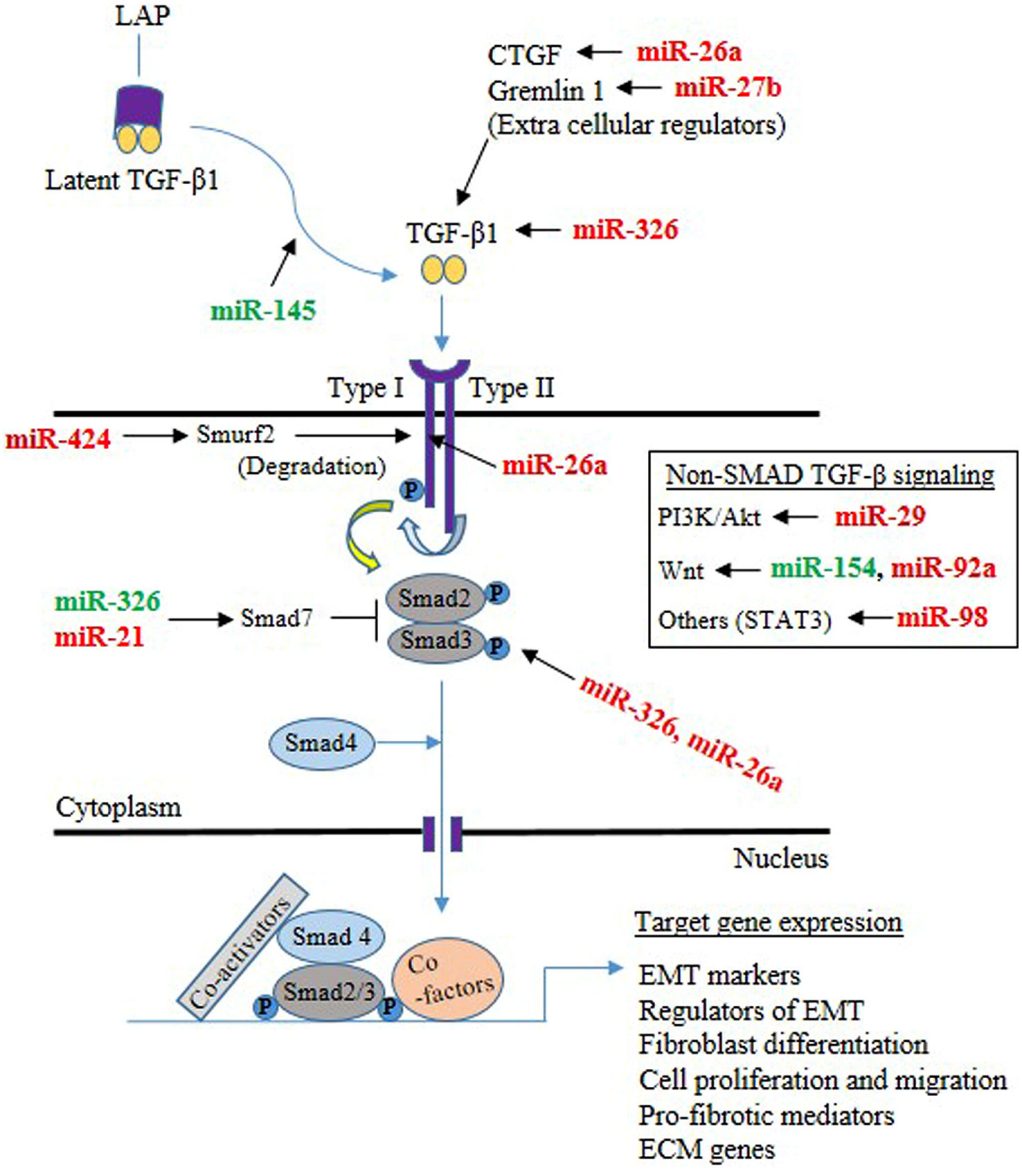
**Mechanisms through which microRNAs (miRNAs) implicated in the TGF-β signaling pathway in the lung epithelial cells and fibroblasts.** The majority of TGF-β is in a latent form and kept inactive by the latency-associated peptide (LAP) in the extracellular milieu. Upon release from LAP, TGF-β dimers then associate with the type II TGF-β receptor, that in turn associates with the type I receptor, leading to the activation of the receptor heterodimer and initiation of a variety of signaling pathways. Both Smad-mediated and non-Smad mediated pathways are involved, leading to activation of target genes involved in epithelial mesenchymal transition (EMT), differentiation, proliferation, migration, pro-fibrotic activity and ECM. miRNA targeting components of TGF-β signaling are shown (Green color indicates enhancer, whereas red color indicates suppressor).

**FIGURE 2:**
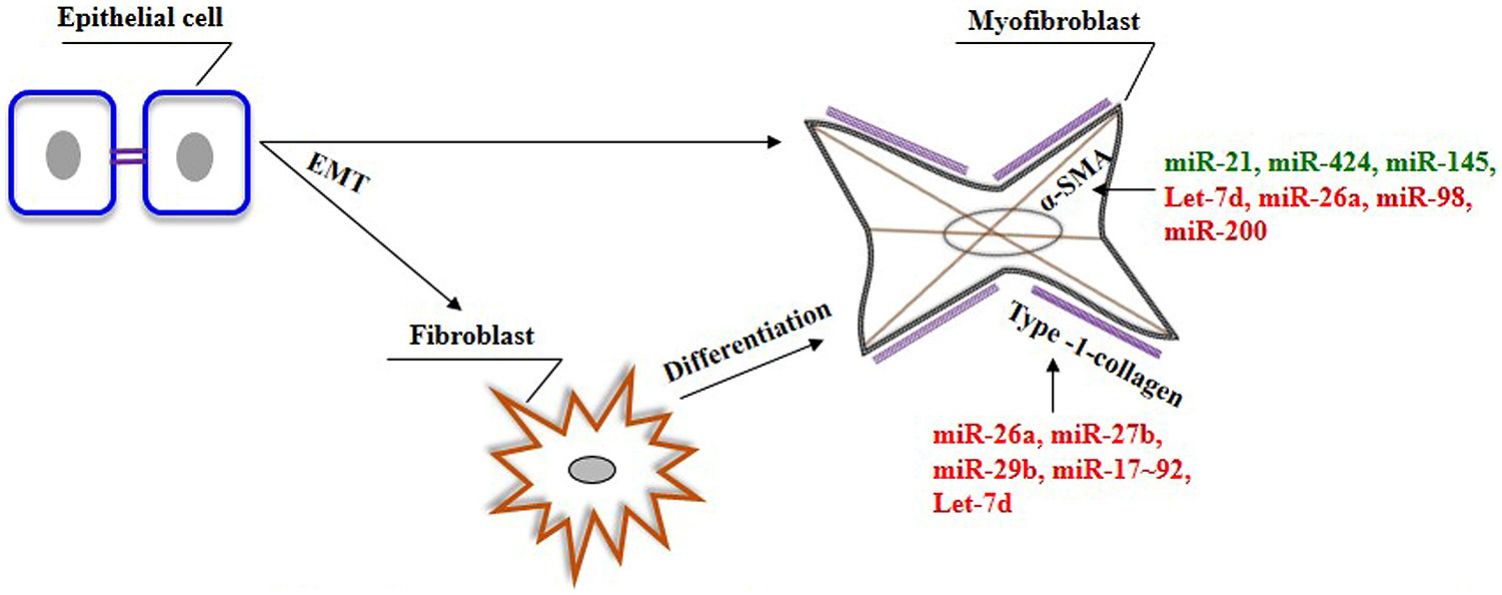
**The role of miRNAs on the genes associated with myofibroblasts phenotype in the lungs.** In response to specific stimuli. epithelial cells can give rise to fibroblasts/myofibroblasts in the lung through a process of EMT. In addition, myofibroblasts can also be derived from resident fibroblasts. Myofibroblasts are characterized by the presence of α-SMA and their ability to produce ECM genes, including type-1-collagen. Green color indicates enhancer, whereas red color indicates suppressor of genes involved in the pathogenic nature of myofibroblasts.

Lung epithelial cells and fibroblasts take a central role in the development of lung fibrosis by undergoing EMT and differentiation, respectively, in response to pro-fibrotic stimuli, resulting in enhanced synthesis of ECM proteins (Sakai and Tager, 2013). TGF-β1, being a prototypical factor for the induction of EMT and fibroblast differentiation, has been extensively used to study the role of miRNAs in lung epithelial cells and fibroblasts. Therefore, this section is focused on the role of miRNAs in lung epithelial cells (see summary in **Table 3**) and fibroblasts (see summary in **Table 4**) upon fibrotic stimuli. Simultaneously, we highlight those miRNAs that are targeting TGF-β signaling events (see **Figure 1**), α-SMA (see **Figure 2**), and COL1 (see **Figure 2**) gene expression during the fibrogenic activity of epithelial cells and fibroblasts.

**Table 3:** Effects of miRNAs in lung epithelial cells.

S. NO	miRNA	Cell type	Species	Regulation on notable target genes and validation method	Putative role	Reference
1	let-7d	A549, RLE-6TN and NHBE	Human and rat	**↓** HMGA2 (qRT-PCR)	Attenuates EMT	Pandit et al., 2010
2	miR-21	Primary alveolar type-2 epithelial cells	Mouse	**↑** Zeb1 and Zeb2 (qRT-PCR)	Promotes EMT	Yamada et al., 2013
3	miR-200	RLE-6TN	Rat	**↓** Zeb1, Zeb2 and Gata3 (qRT-PCR)	Attenuates EMT	Yang et al., 2012
4	miR-29b	A549	Human	**↓** Col1αl and Col3αl (qRT-PCR)	Attenuates EMT	Montgomery et al., 2014
5	miR-326	A549 and NHBE cells	Human	**↓** TGF-β1 (qRT-PCR and ELISA)	Attenuates EMT	Das et al., 2014
6	miR-26a	A549 and mice lungs	Human and mouse	**↓** HMGA2 (Western blot and luciferase assay)	Attenuates EMT	Liang et al., 2014a
7	miR-27b	A549	Human	**↓** Gremlin 1 (qRT-PCR and western blot)	Attenuates EMT	Graham et al., 2014
8	miR-98	A549	Human	**↓** pStat3 and Stat3 (Western blot)	Attenuates EMT	Gao et al., 2014
9	miR-424	A549	Human	**↓** Smurf2 (Western blot)	Promotes EMT	Xiao et al., 2015

**Table 4:** Roles of miRNAs in lung fibroblasts.

S. NO	miRNA	Cell type	Species	Regulation on notable target genes and validation method	Putative role	Reference
1	miR-21	MRC-5	Human	↓ Smad 7 (Western blot)	Promotes fibroblast transdifferentiation	Liu et al., 2010b

2	miR-29	IMR-90	Human	↓ ECM genes (Col5A1, Col5A2, FBN1, LOXL2, FSTL1, PDGFC and SERPINH1), BM related genes (SPARC and Col4A1), Intergrins (ITGA5 and ITGAV), Genes involved in proteolysis and ECM remodeling (ADAM12, ADAM19 and BMP1) and IL-1 pathway (IL1RAP) (mRNA array analysis)	Negative regulators of fibrotic phenotype	Cushing et al., 2011

3	miR-200b, 200c	Primary normal and IPF lung fibroblast	Human and mouse	No specific target defined	Attenuates fibroblast transdifferentiation	Yang et al., 2012

4	miR-154	Primary normal and IPF lung fibroblast	Human	WNT/β-catenin pathway (↑ FZD5, CTNNB1,FZD4,FZD6,KREMEN1, LRP5, WISP1 and β-catenin) (qRT-PCR and western blot)	Promotes proliferation and migration	Milosevic et al., 2012

5	miR-199a-5p	MRC-5	Human	↓ Caveolin-1 (qRT-PCR and western blot)	Pathogenic activation of fibroblast	Lino Cardenas et al., 2013

6	miR-17 ∼ 19 cluster	IPF fibroblast	Human	↓DNMT-1 (Methyl-Profiler DNA Methylation qPCR Primer Assays)	Reverse fibrotic phenotype	Dakhlallah et al., 2013

7	miR-145	MRC-5 and lung fibroblast	Human and mouse	↓ KLF4 (Western blot)	Promotes fibroblast transdifferentiation	Yang et al., 2013a

8	miR-29	IMR90	Human	↓ PI3K-Akt pathway (Western blot)	Reverse fibrotic phenotype	Yang et al., 2013b

9	miR-326	NIH/3T3	Mouse	↓ TGF-β1, SMAD3 and Ets; ↑ (SMAD 7) (ELISA and western blot)	Attenuates fibroblast proliferation and fiobrotic related genes	Das et al., 2014

10	miR-92a	Primary Lung fibroblast	Human	↓ WISP1 (qRT-PCR and ELISA)	Suppresses pro-fibrotic mediator	Berschneider et al., 2014

11	miR-96	Normal and IPF fibroblast	Human	↓ Fox03 (qRT-PCR and western blot)	Promotes proliferation and maintains fibrotic phenotype	Nho et al., 2014

12	miR-26a	MRC-5	Human	↓ pSmad3 and CTGF (Western blot and luciferase assay)	Attenuates fibroblast transdifferentiation	Liang et al., 2014b

13	miR-29b	Primary IPF lung fibroblast	Human	No specific target defined	Reverse fibrotic phenotype	Montgomery et al., 2014

14	miR-210	IPF fibroblast	Human	↓ MNT (Western blot)	Promotes proliferation	Bodempudi et al., 2014

15	miR-26a	Primary fetal lung fibroblast	Human	↓ Cyclin D2, TGF-βR1 and TGF-β2 (qRT-PCR and western blot)	Suppresses proliferation	Li et al., 2014

16	Let-7d	Primary fetal lung fibroblast	Human	↓ HMGA2, SLUG, ID1 and ID2 (Micro array and qRT-PCR)	Reverse fibrotic phenotype	Huleihel et al., 2014

## Effects of miRNAs in Lung Epithelial Cells

Pandit et al. (2010) reported that let-7d inhibition induced a significant increase in the expression of mesenchymal markers such as *N*-cadherin (N-CAD), VIM, and α-SMA in A549, RLE-6TN and normal human bronchial epithelial (NHBE) cells. Overexpression of miR-200 family in RLE-6TN cells, particularly miR-200b and miR-200c, attenuated TGF-β1-induced morphological changes and expression of mesenchymal cell markers, including α-SMA, and VIM (Yang et al., 2012). Further, miR-200 inhibits EMT in RLE-6TN cells by downregulating the expression of EMT promoting transcription factors such as trans-acting T-cell-specific transcription factor GATA-3 (GATA3), and zinc finger E-box-binding homeobox 1/2 (ZEB1 and ZEB2) (Yang et al., 2012). Later, Yamada et al. (2013) reported that the inhibition of miR-21 also attenuated TGF-β1-induced EMT in mouse primary alveolar type II epithelial cells and prevented the expression of VIM, α-SMA and ZEB1/2.

Recently, miR-29b mimic treatment was found to inhibit collagen induction in A549 cells, confirming the ability of miR-29b to block phenotypical changes (Montgomery et al., 2014). Similarly, treatment of A549 and NHBE cells with miR-326 mimics caused a significant downregulation in TGF-β1, which was apparently due to the degradation of TGF-β1 transcripts (Das et al., 2014). Further, anti-miR-326 induced TGF-β1 production and promoted EMT as indicated by increased expression of mesenchymal marker, VIM and decreased expression of epithelial marker, cytokeratin 14 (Das et al., 2014). These results indicate that miR-326 is capable of enforcing epithelial phenotypes by inhibiting TGF-β1.

As miR-26a suppresses HMGA2, a key positive regulator of EMT by binding to its 3′-UTR sequence, inhibition of miR-26a caused the elevation of EMT phenotype in A549 cells and in mice lungs (Liang et al., 2014a,b). These results suggested that loss of miR-26a function could facilitate the transformation of lung epithelial cells into myofibroblasts. Functional screening using a library of miRNA inhibitors identified miR-27b as a direct regulator of COL1 in A549 cells and its inhibition caused a significant increase in COL1 expression (Graham et al., 2014). Subsequently, miR-27b was found to directly target Gremlin 1 by binding to its 3′-UTR and reducing its mRNA levels (Graham et al., 2014). However, TGF-β1 treatment decreased miR-27b expression and caused a corresponding increase in Gremlin 1 level and EMT process, suggesting that TGF-β1 regulates Gremlin1 level by partly modulating the expression of miR-27b (Graham et al., 2014).

A549 cells treated with TGF-β1 morphologically tended to reverse into epithelial cells after intervention with miR-98 (Gao et al., 2014). The expression of α-SMA in miR-98-treated A549 cells was downregulated, whereas the expression of E-CAD was increased, which confirmed the participation of miR-98 in the process of TGF-β1-induced EMT (Gao et al., 2014). The authors also determined that miR-98 treated A549 cells showed significantly reduced STAT3 and p-STAT3 expression following TGF-β1 treatment, which indicated that miR-98 controls TGF-β1-mediated EMT process by regulating STAT3 and p-STAT3 levels in STAT3 pathway.

Upon TGF-β1 treatment, miR-424 expression was found to be increased in A549 cells, which in turn led to increased α-SMA expression (Xiao et al., 2015). miR-424 targets SMAD specific E3 ubiquitin protein ligase 2 (SMURF2), a negative regulator of TGF-β signaling. The combination of miR-424 overexpression and TGF-β1 treatment increased α-SMA and CTGF expression further when compared with the treatment of TGF-β1 alone or miR-424 over-expression alone. The above studies thus suggest that the specific suppression of upregulated miRNAs, such as miR-21 and miR-424, or the specific overexpression of downregulated miRNAs, such as let-7d, miR-200, miR-29b, miR-326, miR-26a, miR-27b and miR-98, may be a viable approach in blocking the excessive EMT process in the fibrotic lungs.

## Roles of miRNAs in Lung Fibroblasts

Transfection of TGF-β1 treated human primary fibroblast cell line, MRC5 with miR-21 precursors induced the transcription of FN and α-SMA, and SMAD2 phosphorylation and decreased SMAD7 expression (Liu et al., 2010b). Thus, miR-21 appears to enhance TGF-β1 signaling events to promote fibrotic phenotype in fibroblasts. Knocking down of miR-29 in TGF-β1 treated human fetal lung fibroblasts (IMR-90) cells upregulates the expression of several collagens, a large number of previously unrecognized ECM-associated and remodeling genes, thus, suggesting the regulatory role of miR-29 over fibrotic related genes in fibroblasts (Cushing et al., 2011). Later, Yang et al. (2013b) observed that treatment of IMR-90 cells with TGF-β1 increased cell proliferation, colony formation and upregulation of COL1. They also concluded that miR-29 mediates anti-fibrogenic effects through downregulation of TGF-β1-induced activation of PI3K-Akt phosphorylation. Consistent with these studies, transfection of miR-29b mimic in IPF fibroblasts was found to control the expression of COL1, in both TGF-β1 treated as well as baseline conditions (Montgomery et al., 2014), further underscoring the anti-fibrotic potential of miR-29 in lung fibroblasts. Further, overexpression of miR-200, particularly miR-200b and miR-200c, markedly attenuated TGF-β1-induced expression of FN and α-SMA in MRC-5 cell line and in lung fibroblasts isolated from mice with experimental pulmonary fibrosis (Yang et al., 2012). In addition, miR-154 was also shown to significantly decrease TGF-β1-induced proliferation of normal human lung fibroblasts (NHLF) and IPF fibroblasts through activation of the WNT/β-catenin pathway (Milosevic et al., 2012).

Further, miR-199a-5p was found to bind to the 3′-UTR of Caveolin 1 (CAV1) mRNA, a negative regulator of TGF-β signaling (Lino Cardenas et al., 2013). In MRC-5 cells, silencing of miR-199a-5p strongly inhibited TGF-β1-mediated differentiation of fibroblasts into myofibroblasts, wound repair and SMAD signaling (Lino Cardenas et al., 2013). These findings demonstrated that miR-199a-5p promotes pathogenic activation of fibroblasts in response to TGF-β1 by regulating CAV1 (Lino Cardenas et al., 2013). Introduction of miR-17∼19 cluster into the fibroblasts derived from IPF patients was found to reduce actin staining to levels similar to the normal fibroblasts and in addition, reduced the expression of several fibrotic related genes such as VEGF, CTGF, COL1a1, and COL13a1 when compared to the vector transfected cells (Dakhlallah et al., 2013). In addition, seed sequences for miR-17, miR-19b, miR-20a, and miR-92a were identified in the 3’-UTR of DNMT-1 mRNA. DNMT-1 expression was found to be increased in IPF fibroblasts when compared to normal fibroblasts. Hence, enhancement of miR-17∼19 cluster expression through suppression of DNMT-1 in IPF fibroblasts could be a novel therapeutic strategy to reverse the fibrotic phenotype (Dakhlallah et al., 2013).

miR-145 was found to promote fibroblast differentiation and its expression was found to be upregulated in TGF-β1-treated human lung fibroblasts (Yang et al., 2013a). Overexpression of miR-145 in human lung fibroblasts increased α-SMA expression, enhanced contractility, and promoted the formation of focal and fibrillary adhesions. It was observed that TGF-β1 upregulates miR-145, which targets KLF4, a known negative regulator of α-SMA, suggesting that miR-145 plays an important role in the differentiation of fibroblasts. On the other hand, miR-326 was found to control TGF-β1 expression, and proliferation and downregulates the expression of pro-fibrotic genes such as ETS1, SMAD3 and MMP-9 in NIH/3T3 cells (Das et al., 2014). It upregulates antifibrotic genes such as SMAD7 in the presence of interleukin-13 (IL-13) and interleukin-1β (IL-1β). These results for the first time suggested that miR-326 acts as an anti-fibrogenic agent in lung fibroblasts. WISP1 has been demonstrated to contribute to IPF pathogenesis and this gene is found to contain target sites for miR-92a in the 3′-UTR region and introduction of miR-92a decreased TGF-β1-induced WISP1 expression (Berschneider et al., 2014). These findings indicated the regulatory role of miR-92a on WISP1 expression in reversing fibrotic phenotype.

FOXO3a is suppressed in IPF fibroblasts, which allows them to expand in this diseased condition. Further, inhibition of miR-96 expression induced FOXO3a mRNA and protein expression, and its target proteins such as p21, p27, and BIM in IPF fibroblasts, resulting in suppression of IPF fibroblast proliferation and promoting their cell death (Nho et al., 2014). Nho et al. (2014) reported the increased expression of miR-96 in IPF fibroblasts inhibits FOXO3a function, causing IPF fibroblasts to maintain their pathological phenotype.

Likewise, anti-fibrogenic role of miR-26a on lung fibroblasts has been established by two recent reports (Li et al., 2014; Liang et al., 2014b). In MRC-5 cells, miR-26a abolished TGF-β1-induced secretion of collagen, and suppressed the expression of fibrotic genes such as COL1, COL4 (type-4-collagen), COL3, α-SMA, SMAD4, and CTGF. In addition, miR-26a inhibited TGF-β1-mediated nuclear translocation of pSMAD3 by directly targeting SMAD4, which determines the nuclear translocation of pSMAD2/SMAD3. As miR-26a blocks the G1/S phase transition through degrading mRNA expression of cyclin-D2 (CCND2) by directly targeting 3′-UTR of cyclin-D2 (CCND2), inhibition of endogenous miR-26a was found to promote proliferation of human lung fibroblasts (Li et al., 2014). These results suggested that miR-26a could suppress TGF-β1-induced proliferation and differentiation of lung fibroblasts. In hypoxia, HIF-2α regulates miR-210 expression and miR-210-mediates proliferation of IPF fibroblasts via repression of its downstream target MNT, a negative regulator of c-MYC (Bodempudi et al., 2014). Hence, hypoxia promotes IPF fibroblast proliferation via stimulating miR-210 expression, which in turn worsens hypoxia.

Lastly, Huleihel et al. (2014) found that let-7d transfection into the lung fibroblasts induced decreased mesenchymal markers expression, phenotypical changes such as reduced proliferation, motility, and a delay in wound healing. Combined let-7d transfection and TGF-β1 treatment resulted in significantly attenuated HMGA2 protein induction by TGF-β1. These results demonstrated that administration of let-7d significantly affects the mesenchymal phenotypic properties of lung fibroblasts. Thus, the above studies clearly demonstrate pro-fibrotic and anti-fibrotic roles of miRNAs on the lung fibroblasts.

## Conclusion and Future Perspectives

Our review outlines the current knowledge on dysregulated miRNAs in the lungs of human IPF as well as their role in animal models of pulmonary fibrosis. In addition, miRNAs that directly regulate pro-inflammatory mediators, EMT, fibroblast proliferation and differentiation, TGF-β signaling (see **Figure 1**), and ECM gene expression (Type-1-collagen) (COL1) (see **Figure 2**) are also discussed. However, there is still plenty of room for improving our understanding about miRNAs and the role that each miR could play in lung pathophysiology.

From the earliest descriptions of patients with pulmonary fibrosis, histological analysis of lung has shown the accumulation of various inflammatory cells such as macrophages, neutrophils, eosinophils and lymphocytes in the interstitium of the lung (Scadding and Hinson, 1967; Crystal et al., 1976). The role of inflammation in IPF has been questioned and de-emphasized over the past few years (Selman et al., 2001; Horowitz and Thannickal, 2006). Although the precise role of inflammation is still unclear, IPF continued to be viewed as a chronic inflammatory disease of the lung parenchyma (Crystal et al., 2002; Barnes and Adcock, 2009; Homer et al., 2011; Wynn, 2011). Due to the influence of miRNA in regulating various cellular functions, attention should be given to understand the role of miRNAs on inflammatory events that drive the onset and progression of pulmonary fibrosis.

Results of *in situ* labeling analysis from several studies have demonstrated the presence of numerous apoptotic epithelial cells in the lung tissues from patients with IPF and in murine models of pulmonary fibrosis (Uhal et al., 1998; Plataki et al., 2005), which is associated with the development of pulmonary fibrosis (Thannickal and Horowitz, 2006). Furthermore, IPF is considered as an age-related disease because two–thirds of IPF patients are older than 66 years at the time of diagnosis (Raghu et al., 2006; Collard, 2010). Thus, IPF is likely to share common pathophysiologic mechanisms of aging such as senescence, deficiencies in DNA repair pathways, telomere shortening, an alteration in DNA methylation pattern, autophagy, mitochondrial dysfunction, stem cell exhaustion and an altered intercellular communication (Thannickal, 2013). Therefore, the interactions between miRNAs and hallmarks of aging that occur in IPF need to be elucidated.

Further, in the lungs of IPF patients, apoptotic positive alveolar epithelial cells colocalize with α-SMA-positive myofibroblasts of foci, indicating the ability of injured epithelial cells to affect the local fibroblast behavior in a paracrine fashion (Uhal et al., 1998). *In vitro* studies have shown that several mediators are responsible for this ability of injured epithelial cells, including TGF-β1 (Morishima et al., 2001), CTGF (Pan et al., 2001) and sonic hedgehog (SHH) (Urase et al., 1996). Similarly, activated fibroblasts are reported to amplify the epithelial apoptosis although the initial cause for epithelial injury in IPF remains elusive. *In vitro* experiments demonstrated that mediators such as angiotensin II and hydrogen peroxide (H_2_O_2_) appear to activate this paracrine action (Uhal et al., 1995; Wang et al., 1999). Hence, further studies are needed to identify the involvement of miRNAs on paracrine interactions of these two cell types in the development of pulmonary fibrosis.

Although the participation of miRNAs in pulmonary fibrosis is evident, the factors that regulate miRNAs in pulmonary fibrosis remain elusive. Pandit et al. (2010) showed that TGF-β1 inhibits let-7d expression, which is mediated through binding of SMAD3 to the let-7d promoter. A recent study revealed the binding of SMAD3 to position 391 bp upstream of the miR-154 gene in TGF-β1 treated cells, but not in the unstimulated cells (Milosevic et al., 2012). In addition, binding of SMAD3 at the 322 bp site upstream of pre-miR-154 was evident either in the presence or absence of TGF-β1 stimulation (Milosevic et al., 2012). However, the mechanisms by which miRNAs are being down or upregulated during IPF warrant more investigations. Furthermore, identifying regulators of miRNA is difficult as more than one mediator or pathways participate in regulating miRNA expression. It is also important to investigate the crosstalk among miRNAs as multiple miRNAs are altered during IPF. Another problem to fully understand target genes of miRNA is that a single miRNA can control hundreds of distinct target genes that potentially affect various cellular pathways. All these unsolved questions require to have additional investigations.

The identification of specific circulating biomarkers for IPF is emphasized in recent time for the potential clinical implications in order to facilitate diagnosis and prediction of disease progression (Yang et al., 2015). The importance of having such biomarkers in IPF was recently reviewed by Cicchitto and Sanguinetti (2013). Several reports suggested that miRNAs are secreted as micro vesicles or exosome and apoptotic bodies, and hence they are stable and abundant and can be readily detected in the circulation (Rayner and Hennessy, 2013). In the context of miRNAs as circulating biomarkers, indeed, in mice, Huang et al. (2012) reported that miR-125b-5p, miR-128, miR-30e, and miR-20b were significantly altered in the lung tissue and in plasma of smoking-induced pulmonary fibrosis. Li et al. (2013) reported that serum miR-21 expression was significantly higher in IPF patients than in healthy controls and is associated with the severity of tissue damage as indicated by Forced vital capacity (FVC) and radiologic examinations. Further, Wang et al. (2014) identified plasma miR-1229-3p, miR-145-5p, miR-338-3p, mIR-3620-3p, miR-4485, miR-4707-3p, and miR-636 as promising biomarkers of chronic obstructive pulmonary disease (COPD). Hence, given the important roles of miRNAs in IPF, identification of more circulatory miRNAs will likely increase the use of miRNA as potential biomarkers for the early diagnosis of pulmonary fibrosis.

Regarding the use of miRNA as a target for therapeutic tool, several approaches may be used to control pathological miRNA dysregulation. The upregulated miRNAs could be optimally managed through the usage of antagomirs, locked nucleic acid (LAN) anti-miR, miRNA sponge, and miR-masks. Conversely, the low expressed miRNAs could be restored through molecular strategies such as mimic miRNA or adenovirus associated vectors (AAVs) carrying miRNA encoding gene. Furthermore, use of miRNAs as *in vivo* therapeutic agents is attractive, but faces considerable challenges, including non-specific targets, tissue-specific delivery, and activation of the innate and adaptive immune responses. However, future efforts may lead to the development of novel therapeutic approaches targeting miRNAs for this incurable and often devastating disorder.

## Conflict of Interest Statement

The authors declare that the research was conducted in the absence of any commercial or financial relationships that could be construed as a potential conflict of interest.
